# Supplementary material for: Obesogenic Diet in Mice Leads to Inflammation and Oxidative Stress in the Mother in Association with Sex-Specific Changes in Fetal Development, Inflammatory Markers and Placental Transcriptome
Source: Antioxidants (Basel). 2024 Mar 28;13(4):411. doi: 10.3390/antiox13040411 (PMC11047652; doi:10.3390/antiox13040411)
Supplement: Supplementary file 1 [file antioxidants-13-00411-s001.zip › antioxidants-2887517-supplementary.pdf]

Supplementary Materials

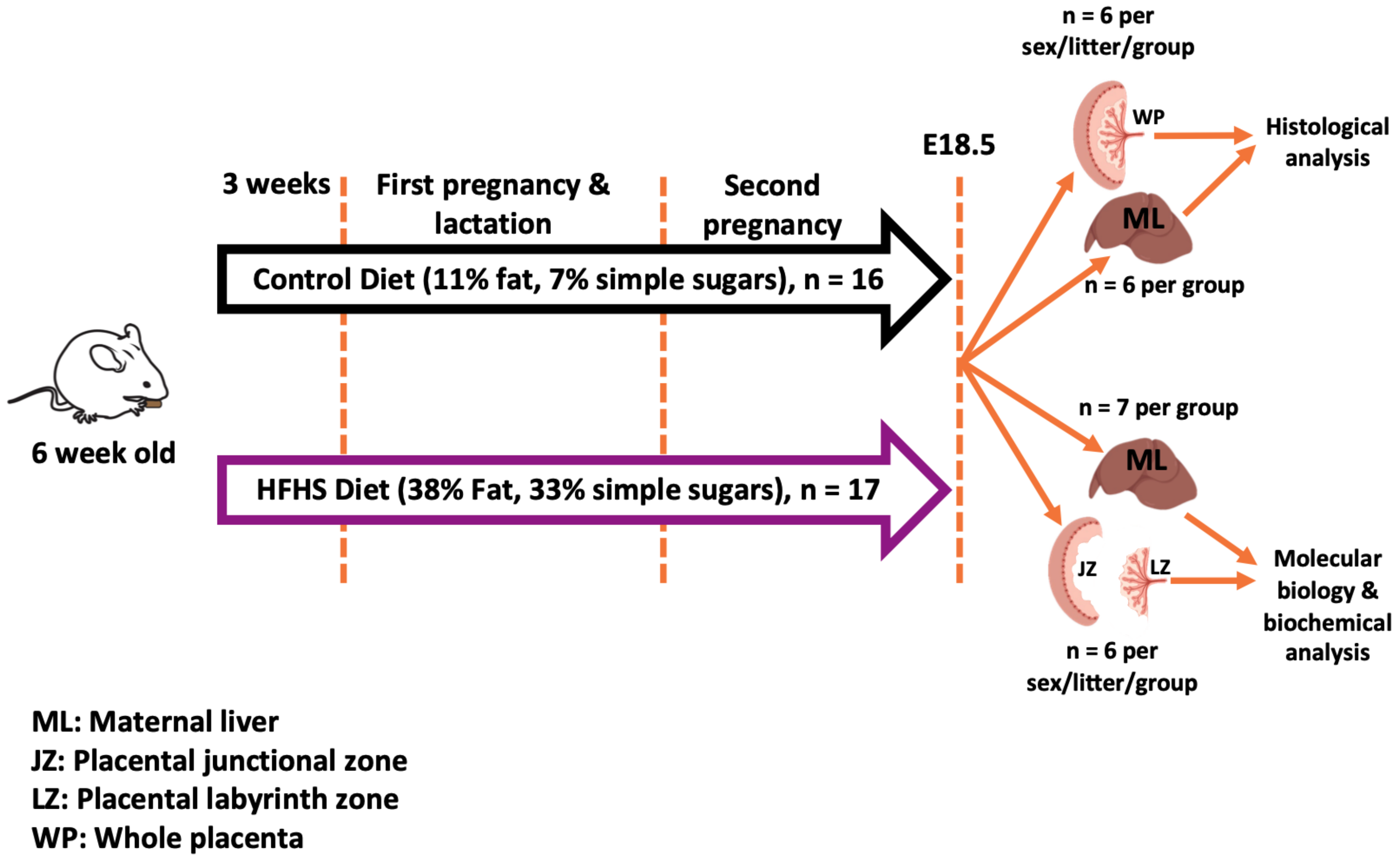

Figure S1. Study design.

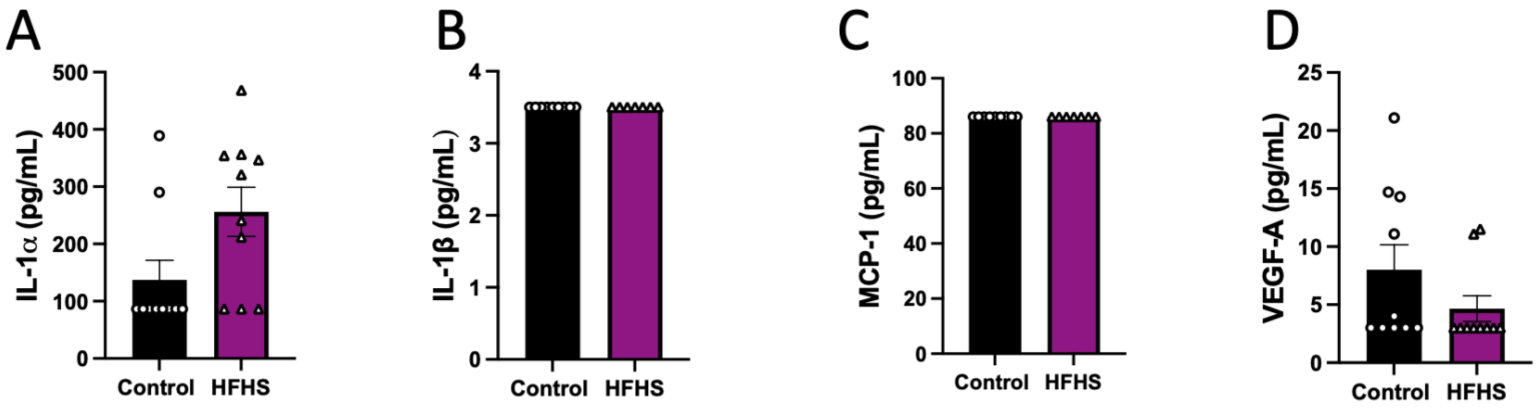

Figure S2. Maternal cytokines below detectable levels of the assay.

Plasma maternal levels of IL-1α (A), IL-β (B), MCP-1 (C), and VEGF-A (D). Groups are controlled (Control, black bars, circles n=10) and high-fat high-sugar diet (HFHS, magenta bars, triangles, n=10). Statistical analysis was not performed due to the under-detection of most of the cytokines.

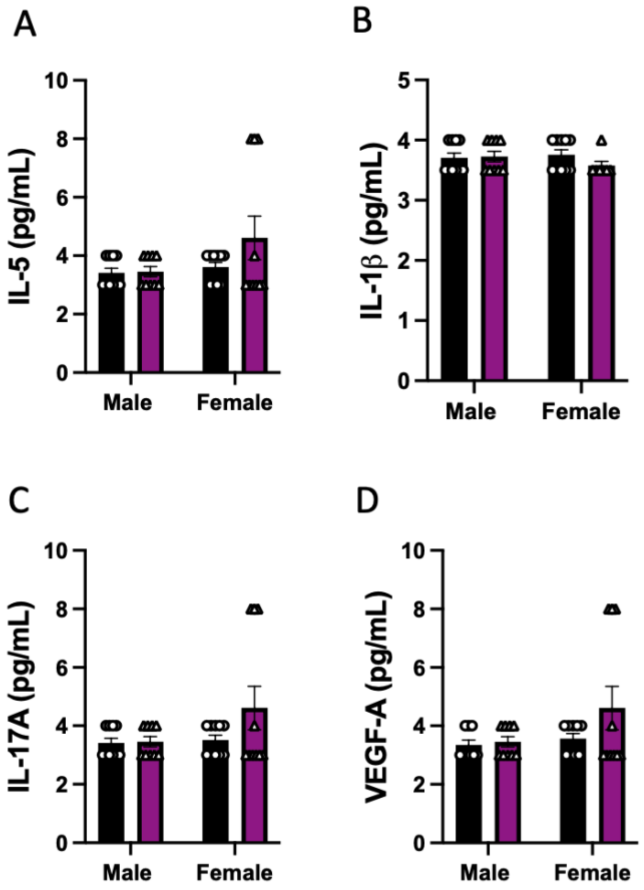

Figure S3. Fetal cytokines below detectable levels of the assay.

Plasma fetal levels of IL-5 (A), IL-β (B), IL-17A (C) and VEGF-A (D). Groups are controlled (Control, black bars, circles n=10) and high-fat high-sugar diet (HFHS, magenta bars, triangles, n=10). Statistical analysis was not performed due to the under-detection of most of the cytokines.

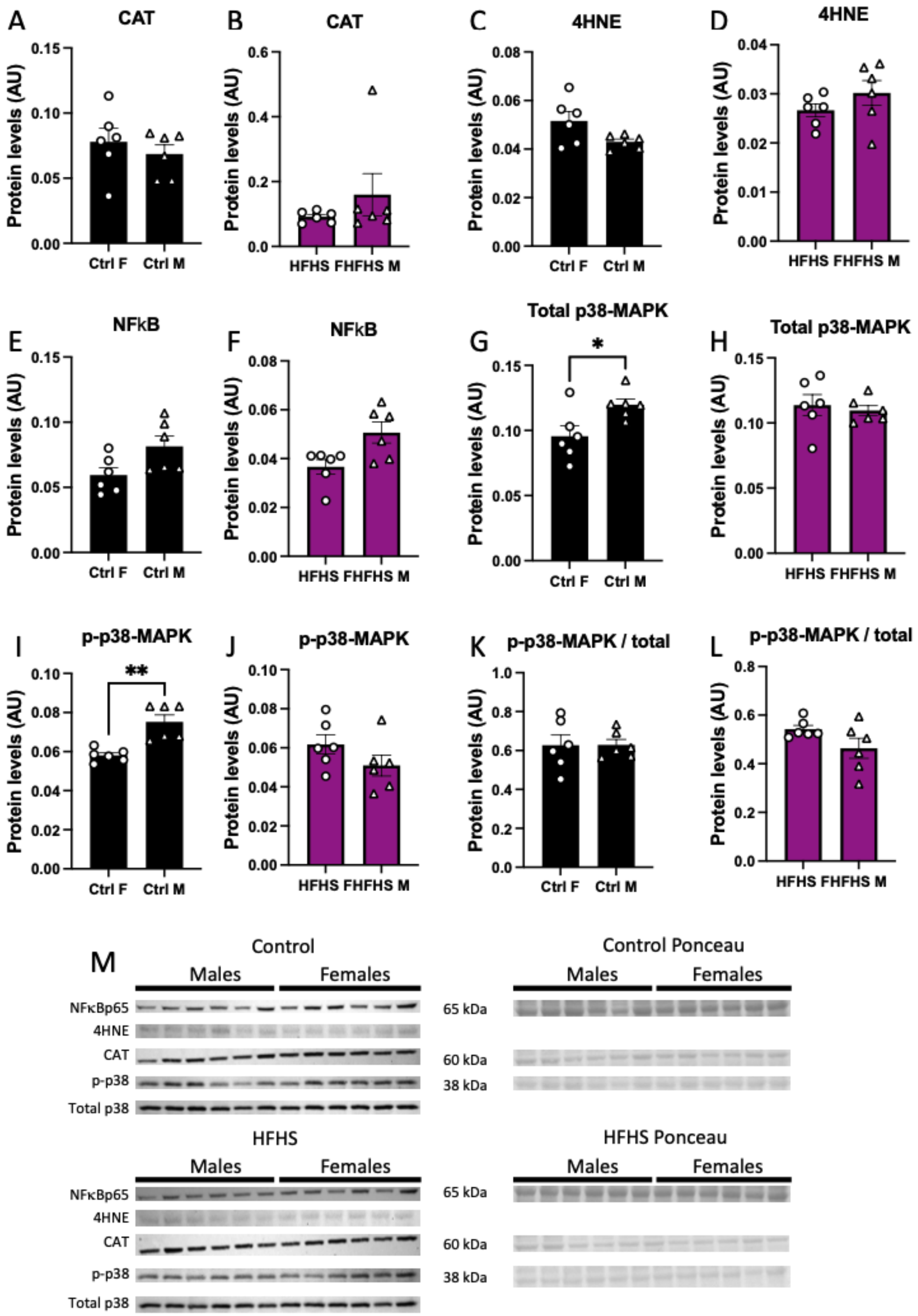

A
CAT
B
CAT
C
4HNE
D
4HNE
E
NFkB
F
NFkB
G
Total p38-MAPK
H
Total p38-MAPK
I
p-p38-MAPK
J
p-p38-MAPK
K
p-p38-MAPK / total
L
p-p38-MAPK / total
Protein levels (AU)
Ctrl F
Ctrl M
HFHS F
HFHS M
M
Control
Control Ponceau
HFHS
HFHS Ponceau
Males
Females
NFκBp65
4HNE
CAT
p-p38
Total p38
65 kDa
60 kDa
38 kDa

Figure S4. Placental labyrinth zone protein levels – Comparison by sex.

Protein levels of catalase (CAT, A-B), 4-Hydroxynonenal (4-HNE, C-D), total levels of p65-NFκB (E-F), total p38 (G-H), phosphorylated p38 (I-J), phosphorylated to total p38 (K-L) and representative image of western blots (M) from placental labyrinth zone of control (Ctrl F, Ctrl M,) and HFHS (HFHS M, HFHS M) are shown. Groups are control (Control, black bars, circles, n=6) and high-fat high-sugar diet (HFHS, magenta bars, triangles, n=6). Each dot represents one individual. Mean ± SEM is shown. Rout test was applied to identify outliers and Shapiro-Wilk to determine the normality of data. Data was submitted to the t-Student test or Mann-Whitney according to data distribution. *$p \leq 0.05$ vs. female; **$p \leq 0.005$ vs. female.

Table S1. Gene Identification

Gene ID	Gene Symbol
ENSMUSG00000026051	1500015O10Rik
ENSMUSG00000051606	2010001K21Rik
ENSMUSG00000085287	4833418N02Rik
ENSMUSG00000025783	4930412O13Rik
ENSMUSG00000097494	4933406C10Rik
ENSMUSG00000101848	4933417E11Rik
ENSMUSG00000110140	5430421F17Rik
ENSMUSG00000113831	AC163032.1
ENSMUSG00000032281	Acsbg1
ENSMUSG00000044017	Adgrd1
ENSMUSG00000015452	Ager
ENSMUSG00000033715	Akr1c14
ENSMUSG00000039062	Anpep
ENSMUSG00000044338	Aplnr
ENSMUSG00000090698	Apold1
ENSMUSG00000038204	Asb10
ENSMUSG00000039200	Atf7ip2
ENSMUSG00000039347	Atp6v0e2
ENSMUSG00000079065	BC005561
ENSMUSG00000112160	BC024063
ENSMUSG00000105345	BC030343
ENSMUSG00000028167	Bdh2
ENSMUSG00000095742	CAAA01147332.1
ENSMUSG00000020866	Cacna1g
ENSMUSG00000054793	Cadm4
ENSMUSG00000054083	Capn12
ENSMUSG00000040828	Catsperd
ENSMUSG00000045790	Ccdc149
ENSMUSG00000039155	Cdh26
ENSMUSG00000034918	Cdhr2
ENSMUSG00000040809	Chil3
ENSMUSG00000037594	Clba1
ENSMUSG00000063564	Col23a1
ENSMUSG00000004415	Col26a1
ENSMUSG00000036731	Cysrt1
ENSMUSG00000031970	Dbndd1
ENSMUSG00000028078	Dclk2
ENSMUSG00000059327	Eda
ENSMUSG00000048988	Elfn1
ENSMUSG00000032262	Elovl4
ENSMUSG00000022468	Endou
ENSMUSG00000028024	Enpep
ENSMUSG00000113346	Eprn
ENSMUSG00000037709	Fam13a
ENSMUSG00000078670	Fam174b
ENSMUSG00000025854	Fam20c
ENSMUSG00000025738	Fbxl16
ENSMUSG00000024827	Gldc
ENSMUSG00000087086	Gm13269
ENSMUSG00000079009	Gm14139
ENSMUSG00000085524	Gm14224
ENSMUSG00000090015	Gm15446
ENSMUSG00000107181	Gm17768
ENSMUSG00000093507	Gm20627
ENSMUSG00000079323	Gm20661
ENSMUSG00000090625	Gm20721
ENSMUSG00000092225	Gm2381
ENSMUSG00000099143	Gm27483
ENSMUSG00000098816	Gm27786
ENSMUSG00000113252	Gm33489
ENSMUSG00000102411	Gm36936
ENSMUSG00000103050	Gm38273
ENSMUSG00000112474	Gm46209
ENSMUSG00000112922	Gm47572
ENSMUSG00000115593	Gm4824
ENSMUSG00000112963	Gm6093
ENSMUSG00000113428	Gm7664
ENSMUSG00000114155	Gm7986
ENSMUSG00000089901	Gm8113
ENSMUSG00000046733	Gprc5a
ENSMUSG00000050440	Hamp
ENSMUSG00000056758	Hmga2
ENSMUSG00000029919	Hpgds
ENSMUSG00000001249	Hpn
ENSMUSG00000023046	Igfbp6
ENSMUSG00000095079	Igha
ENSMUSG00000076609	Igkc
ENSMUSG00000006538	Ihh
ENSMUSG00000027399	Il1a
ENSMUSG00000030330	Ing4

ENSMUSG00000050671	Ism2
ENSMUSG00000067149	Jchain
ENSMUSG00000025221	Kcnip2
ENSMUSG00000002908	Kcnn1
ENSMUSG00000050463	Krt78
ENSMUSG00000015882	Lcorl
ENSMUSG00000050966	Lin28a
ENSMUSG00000078952	Lncenc1
ENSMUSG00000025185	Loxl4
ENSMUSG00000045045	Lrfn4
ENSMUSG00000001802	Lrp3
ENSMUSG00000040533	Matn1
ENSMUSG00000053702	Nebl
ENSMUSG00000036902	Neto2
ENSMUSG00000042988	Notum
ENSMUSG00000051228	Nyx
ENSMUSG00000021390	Ogn
ENSMUSG00000029718	Pcolce
ENSMUSG00000000489	Pdgfb
ENSMUSG00000032105	Pdzd3
ENSMUSG00000028716	Pdzk1ip1
ENSMUSG00000027674	Pex5l
ENSMUSG00000024738	Pga5
ENSMUSG00000041801	Phlda3
ENSMUSG00000040543	Pitpnm3
ENSMUSG00000021506	Pitx1
ENSMUSG00000056220	Pla2g4a
ENSMUSG00000003282	Plag1
ENSMUSG00000045731	Pnoc
ENSMUSG00000005268	Prlr
ENSMUSG00000093565	Rab26os
ENSMUSG00000049404	Rarres1
ENSMUSG00000027347	Rasgrp1
ENSMUSG00000034209	Rasl10a
ENSMUSG00000037962	Rflna
ENSMUSG00000024206	Rfx2
ENSMUSG00000038530	Rgs4
ENSMUSG00000081895	Rpl17-ps10
ENSMUSG00000096699	Rps19-ps4
ENSMUSG00000047735	Samd9l
ENSMUSG00000046229	Scand1
ENSMUSG00000025203	Scd2
ENSMUSG00000007279	Scube2
ENSMUSG00000040569	Slc26a7
ENSMUSG00000035699	Slc51a
ENSMUSG00000031089	Slc6a14
ENSMUSG00000022180	Slc7a8
ENSMUSG00000037994	Slc9b2
ENSMUSG00000001761	Smo
ENSMUSG00000108414	Snhg1
ENSMUSG00000027488	Snta1
ENSMUSG00000053030	Spink2
ENSMUSG00000036928	Stag3
ENSMUSG00000038576	Susd4
ENSMUSG00000046694	Tent5b
ENSMUSG00000035686	Thrsp
ENSMUSG00000091898	Tnnc1
ENSMUSG00000021573	Tppp
ENSMUSG00000014846	Tppp3
ENSMUSG00000056596	Trnp1
ENSMUSG00000026202	Tuba4a
ENSMUSG00000031380	Vegfd
ENSMUSG00000068231	Vmn1r43
ENSMUSG00000025082	Vwa2
ENSMUSG00000037989	Wnk2
ENSMUSG00000036856	Wnt4
ENSMUSG00000074578	Zfas1
ENSMUSG00000029813	1600015I10Rik
ENSMUSG00000071103	1700029J07Rik
ENSMUSG00000055833	1700034H15Rik
ENSMUSG00000027327	1700037H04Rik
ENSMUSG00000097412	1810014B01Rik
ENSMUSG00000020812	1810032O08Rik
ENSMUSG00000100680	1810044D09Rik
ENSMUSG00000042041	2010003K11Rik
ENSMUSG00000063522	2010109I03Rik
ENSMUSG00000097536	2610037D02Rik
ENSMUSG00000101731	3830432H09Rik
ENSMUSG00000107402	4732416N19Rik
ENSMUSG00000090031	4732440D04Rik
ENSMUSG00000072762	4930522L14Rik
ENSMUSG00000072893	4933439C10Rik
ENSMUSG00000116145	5730521K06Rik
ENSMUSG00000097375	6720427I07Rik
ENSMUSG00000072982	9230009I02Rik
ENSMUSG00000038884	A230050P20Rik
ENSMUSG00000096929	A330023F24Rik

ENSMUSG00000097124	A530020G20Rik
ENSMUSG00000101013	A630072M18Rik
ENSMUSG00000116673	A630089N07Rik
ENSMUSG00000032735	Ablim3
ENSMUSG00000032724	Abtb2
ENSMUSG00000116594	AC133488.1
ENSMUSG00000116813	AC140300.3
ENSMUSG00000116908	AC154232.2
ENSMUSG00000117333	AC154486.3
ENSMUSG00000111394	AC160637.1
ENSMUSG00000030935	Acsm3
ENSMUSG00000040537	Adam22
ENSMUSG00000018500	Adora2b
ENSMUSG00000090086	AI480526
ENSMUSG00000078247	Airn
ENSMUSG00000061603	Akap6
ENSMUSG00000063810	Alms1
ENSMUSG00000037907	Ankrd13b
ENSMUSG00000050914	Ankrd37
ENSMUSG00000005681	Apoa2
ENSMUSG00000002985	Apoe
ENSMUSG00000038295	Atg9b
ENSMUSG00000108461	AV356131
ENSMUSG00000085133	B930095G15Rik
ENSMUSG00000040605	Bace2
ENSMUSG00000051444	Bbs12
ENSMUSG00000060149	BC002059
ENSMUSG00000000706	Btn1a1
ENSMUSG00000063623	C230062I16Rik

ENSMUSG00000063897	CAAA01118383.1
ENSMUSG00000006056	Calcoco2
ENSMUSG00000038128	Camk4
ENSMUSG00000049676	Catsperg1
ENSMUSG00000040525	Cblc
ENSMUSG00000028689	Ccdc163
ENSMUSG00000029875	Ccdc184
ENSMUSG00000043923	Ccdc84
ENSMUSG00000047810	Ccdc88b
ENSMUSG00000027715	Ccna2
ENSMUSG00000026012	Cd28
ENSMUSG00000032595	Cdhr4
ENSMUSG00000056501	Cebpb
ENSMUSG00000060402	Chst8
ENSMUSG00000023906	Cldn6
ENSMUSG00000030157	Clec2d
ENSMUSG00000026034	Clk1
ENSMUSG00000020953	Coch
ENSMUSG00000058806	Col13a1
ENSMUSG00000028626	Col9a2
ENSMUSG00000027570	Col9a3
ENSMUSG00000025196	Cpn1
ENSMUSG00000024008	Cpne5
ENSMUSG00000036596	Cpz
ENSMUSG00000027230	Creb3l1
ENSMUSG00000035041	Creb3l3
ENSMUSG00000023272	Creld2
ENSMUSG00000106106	CT010467.1
ENSMUSG00000052336	Cx3cr1

ENSMUSG00000048065	Cyb5r2
ENSMUSG00000106820	D5Ertd605e
ENSMUSG00000007039	Ddah2
ENSMUSG00000015377	Dennd6b
ENSMUSG00000048489	Depp1
ENSMUSG00000009092	Derl3
ENSMUSG00000009145	Dqx1
ENSMUSG00000040415	Dtx3
ENSMUSG00000028601	Echdc2
ENSMUSG00000003410	Elavl3
ENSMUSG00000029675	Eln
ENSMUSG00000028664	Ephb2
ENSMUSG00000050248	Evc2
ENSMUSG00000052125	F730043M19Rik
ENSMUSG00000033799	Fam208b
ENSMUSG00000020614	Fam20a
ENSMUSG00000024598	Fbn2
ENSMUSG00000021974	Fgf9
ENSMUSG00000085396	Firre
ENSMUSG00000020635	Fkbp1b
ENSMUSG00000047787	Flrt1
ENSMUSG00000041559	Fmod
ENSMUSG00000008200	Fnbp4
ENSMUSG00000097694	G730013B05Rik
ENSMUSG00000024462	Gabbr1
ENSMUSG00000031340	Gabre
ENSMUSG00000023267	Gabrr2
ENSMUSG00000096954	Gdap10
ENSMUSG00000030703	Gdpd3

ENSMUSG00000048582	Gja3
ENSMUSG00000057123	Gja5
ENSMUSG00000031163	Glod5
ENSMUSG00000060730	Gm10086
ENSMUSG00000066116	Gm10154
ENSMUSG00000073985	Gm10602
ENSMUSG00000097773	Gm10614
ENSMUSG00000074284	Gm10658
ENSMUSG00000085152	Gm11496
ENSMUSG00000072915	Gm12258
ENSMUSG00000087142	Gm12454
ENSMUSG00000100514	Gm12960
ENSMUSG00000085767	Gm13563
ENSMUSG00000082196	Gm14231
ENSMUSG00000081865	Gm15484
ENSMUSG00000085917	Gm15899
ENSMUSG00000086287	Gm15972
ENSMUSG00000087381	Gm16008
ENSMUSG00000089769	Gm16574
ENSMUSG00000097042	Gm17491
ENSMUSG00000101599	Gm20342
ENSMUSG00000065767	Gm23849
ENSMUSG00000076258	Gm23935
ENSMUSG00000097730	Gm26588
ENSMUSG00000101603	Gm28730
ENSMUSG00000096218	Gm2916
ENSMUSG00000109179	Gm35339
ENSMUSG00000079584	Gm364
ENSMUSG00000112991	Gm36635
ENSMUSG00000104344	Gm38077
ENSMUSG00000103509	Gm38372
ENSMUSG00000108010	Gm38708
ENSMUSG00000106022	Gm42929
ENSMUSG00000105677	Gm43328
ENSMUSG00000107096	Gm43597
ENSMUSG00000104546	Gm43858
ENSMUSG00000107876	Gm43936
ENSMUSG00000109390	Gm45718
ENSMUSG00000109836	Gm45884
ENSMUSG00000097750	Gm4673
ENSMUSG00000114185	Gm47662
ENSMUSG00000109378	Gm49396
ENSMUSG00000024869	Gm49405
ENSMUSG00000111290	Gm5122
ENSMUSG00000098050	Gm5345
ENSMUSG00000062461	Gm5453
ENSMUSG00000108366	Gm5586
ENSMUSG00000101834	Gm5829
ENSMUSG00000097937	Gm6967
ENSMUSG00000099843	Gm7160
ENSMUSG00000105408	Gm7285
ENSMUSG00000055771	Gm7936
ENSMUSG00000062458	Gm8623
ENSMUSG00000097906	Gm9625
ENSMUSG00000115483	Gm9732
ENSMUSG00000048240	Gng7
ENSMUSG00000039720	Got1l1
ENSMUSG00000029510	Gpc2
ENSMUSG00000055653	Gpc3
ENSMUSG00000058396	Gpr182
ENSMUSG00000045281	Gpr20
ENSMUSG00000021095	Gsc
ENSMUSG00000107369	Gstm2-ps1
ENSMUSG00000041624	Gucy1a2
ENSMUSG00000027360	Hdc
ENSMUSG00000039307	Hexdc
ENSMUSG00000069270	Hist1h2ac
ENSMUSG00000047246	Hist1h2be
ENSMUSG00000068854	Hist2h2be
ENSMUSG00000017950	Hnf4a
ENSMUSG00000001655	Hoxc13
ENSMUSG00000078915	Hsp25-ps1
ENSMUSG00000020048	Hsp90b1
ENSMUSG00000026864	Hspa5
ENSMUSG00000006205	Htra1
ENSMUSG00000031838	Ifi30
ENSMUSG00000020429	Igfbp1
ENSMUSG00000070427	Il18bp
ENSMUSG00000056899	Immp2l
ENSMUSG00000035270	Impg2
ENSMUSG00000037206	Islr
ENSMUSG00000090210	Itga10
ENSMUSG00000026768	Itga8
ENSMUSG00000087149	Itih5l-ps
ENSMUSG00000055862	Izumo4
ENSMUSG00000048534	Jaml
ENSMUSG00000079056	Kcnip3

ENSMUSG00000046410	Kcnk6
ENSMUSG00000067750	Khdc1a
ENSMUSG00000004187	Kifc2
ENSMUSG00000038056	Kmt2c
ENSMUSG00000035545	Leng8
ENSMUSG00000043501	Lgals2
ENSMUSG00000053964	Lgals4
ENSMUSG00000053846	Lipg
ENSMUSG00000025500	Lmntd2
ENSMUSG00000009356	Lpo
ENSMUSG00000036957	Lrfn3
ENSMUSG00000026443	Lrrn2
ENSMUSG00000001870	Ltbp1
ENSMUSG00000002020	Ltbp2
ENSMUSG00000024940	Ltbp3
ENSMUSG00000020377	Ltc4s
ENSMUSG00000034041	Lyl1
ENSMUSG00000030041	M1ap
ENSMUSG00000031303	Map3k15
ENSMUSG00000021268	Meg3
ENSMUSG00000041921	Metap1d
ENSMUSG00000002274	Metrn
ENSMUSG00000097391	Mirg
ENSMUSG00000041445	Mmrn2
ENSMUSG00000073414	Mpig6b
ENSMUSG00000029918	Mrps33
ENSMUSG00000007035	Msh5
ENSMUSG00000021815	Mss51
ENSMUSG00000031200	Mtcp1
ENSMUSG00000057280	Musk
ENSMUSG00000046916	Myct1
ENSMUSG00000034906	Ncaph
ENSMUSG00000023087	Noct
ENSMUSG00000030562	Nox4
ENSMUSG00000022421	Nptxr
ENSMUSG00000029605	Oas1b
ENSMUSG00000047094	Ofcc1
ENSMUSG00000034160	Ogt
ENSMUSG00000027848	Olfml3
ENSMUSG00000051048	P4ha3
ENSMUSG00000022194	Pabpn1
ENSMUSG00000037196	Pacrg
ENSMUSG00000032278	Paqr5
ENSMUSG00000022033	Pbk
ENSMUSG00000038370	Pcp4l1
ENSMUSG00000020131	Pcsk4
ENSMUSG00000023987	Pgc
ENSMUSG00000031295	Phka2
ENSMUSG00000010760	Phlda2
ENSMUSG00000024011	Pi16
ENSMUSG00000082286	Pisd-ps1
ENSMUSG00000033526	Ppip5k1
ENSMUSG00000015474	Ppt2
ENSMUSG00000019756	Prl8a1
ENSMUSG00000027376	Prom2
ENSMUSG00000074925	Ptar1
ENSMUSG00000034117	Ptgdr2
ENSMUSG00000028036	Ptgfr
ENSMUSG00000045826	Ptprcap
ENSMUSG00000026204	Ptprn
ENSMUSG00000029449	Rhof
ENSMUSG00000097451	Rian
ENSMUSG00000028139	Riiad1
ENSMUSG00000037386	Rims2
ENSMUSG00000116090	Rpl19-ps6
ENSMUSG00000051723	Rpl31-ps13
ENSMUSG00000044199	S1pr4
ENSMUSG00000022769	Sdf2l1
ENSMUSG00000057969	Sema3b
ENSMUSG00000058153	Sez6l
ENSMUSG00000029321	Slc10a6
ENSMUSG00000023829	Slc22a1
ENSMUSG00000022199	Slc22a17
ENSMUSG00000034248	Slc25a37
ENSMUSG00000085028	Slc2a4rg-ps
ENSMUSG00000026791	Slc2a8
ENSMUSG00000005802	Slc30a4
ENSMUSG00000011034	Slc5a1
ENSMUSG00000030769	Slc5a11
ENSMUSG00000014786	Slc9a5
ENSMUSG00000050150	Slc9b1
ENSMUSG00000085385	Snhg17
ENSMUSG00000086859	Snhg20
ENSMUSG00000085241	Snhg3
ENSMUSG00000080316	Spaca6
ENSMUSG00000020867	Spata20
ENSMUSG00000033594	Spata2l

ENSMUSG00000051457	Spn
ENSMUSG00000067889	Sptbn2
ENSMUSG00000071112	Spx
ENSMUSG00000060445	Sycp2
ENSMUSG00000031098	Syt8
ENSMUSG00000031939	Taf1d
ENSMUSG00000052031	Tagap1
ENSMUSG00000021187	Tc2n
ENSMUSG00000009628	Tex15
ENSMUSG00000078922	Tgtp1
ENSMUSG00000034771	Tle2
ENSMUSG00000024245	Tmem178
ENSMUSG00000042854	Trp53rkb
ENSMUSG00000029868	Trpv6
ENSMUSG00000039021	Ttc16
ENSMUSG00000085873	Ttc39aos1
ENSMUSG00000030276	Ttll3
ENSMUSG00000032596	Uba7
ENSMUSG00000043592	Unc5cl
ENSMUSG00000034911	Ushbp1
ENSMUSG00000022382	Wnt7b
ENSMUSG00000073125	Xlr3b
ENSMUSG00000067768	Xlr4b
ENSMUSG00000031362	Xlr4c
ENSMUSG00000034265	Zdhhc14
ENSMUSG00000034163	Zfc3h1
ENSMUSG00000074731	Zfp345
ENSMUSG00000000552	Zfp385a
ENSMUSG00000024276	Zfp397
ENSMUSG00000061371	Zfp873
ENSMUSG00000030424	Zfp939
ENSMUSG00000096696	Zfp960
ENSMUSG00000078866	Zfp970
ENSMUSG00000034949	Zfr2
ENSMUSG00000073062	Zxdb

Table S2. Biological process of interest for genes upregulated by a maternal HFHS diet (overall, female-specific and male-specific comparisons). Biological processes determined by the DAVID analysis tool and the comparison when altered are shown for upregulated genes related to immune response, lipid or fatty acid metabolic process, response to hypoxia & ROS, iron transport, nutrients transport, and angiogenesis. Data are n = 12 per dietary group for overall and n = 6 per group for sex-specific comparisons.

	Biological process related to:						Altered when comparing Ctrl vs HFHS:		
Upregulated DEGs	Immune response	Lipid or fatty acid metabolic process	Response to hypoxia & ROS	Ion transport	Nutrients transport	Angiogenesis	Overall	Females	Males
Chil3	x						Yes	Yes	Yes
Pla2g4a	x	x					Yes	Yes	Yes
Slc9b2				x			Yes	Yes	Yes
Bdh2		x					Yes	Yes	No
Slc26a7				x			Yes	Yes	No
Slc6a14				x			Yes	Yes	No
Cd28	x						Yes	No	No
Rasgrp1	x						Yes	No	Yes
Tgtp1	x						Yes	No	No
Hamp	x			x			Yes	No	Yes
Il1a	x						Yes	No	Yes
Snhg1	x						Yes	No	Yes
Apold1		x	x		x	x	Yes	No	Yes
Cyb5r2		x					Yes	No	Yes

Gdpd3		x					Yes	No	No
Hpgds		x					Yes	No	Yes
Acsbg1		x					Yes	No	Yes
Acsm3		x					Yes	No	No
Nox4			x				Yes	No	No
Gucy1a2			x				Yes	No	No
Ptprn			x				Yes	No	No
Rgs4				x			Yes	No	Yes
Slc10a6				x			Yes	No	No
Fgf9						x	Yes	No	No
Slc30a4				x			No	Yes	No
Slc9b1				x			No	Yes	No
Oas1b	x						No	No	Yes
Clec2d	x						No	No	Yes
Gdap10	x						No	No	Yes
Il18bp	x						No	No	Yes
Lgals4	x						No	No	Yes
Pisd-ps1	x						No	No	Yes
Snhg20	x						No	No	Yes
Alms1		x					No	No	Yes
Echdc2		x					No	No	Yes
Phka2		x					No	No	Yes
Gabre				x			No	No	Yes
Gabrr2				x			No	No	Yes
Atg9b					x		No	No	Yes
Spx					x		No	No	Yes

Table S3. Biological process of interest for genes downregulated by a maternal HFHS diet (overall, female-specific and male-specific comparisons). Biological processes determined by the DAVID analysis tool and the comparison when altered are shown for downregulated genes related to immune response, lipid or fatty acid metabolic process, response to hypoxia & ROS, iron transport, nutrients transport, and angiogenesis. Data are n = 12 per dietary group for overall and n = 6 per group for sex-specific comparisons.

	Biological process related to:						Altered when comparing Ctrl vs HFHS:		
Upregulated DEGs	Immune response	Lipid or fatty acid metabolic process	Response to hypoxia & ROS	Ion transport	Nutrients transport	Angiogenesis	Overall	Females	Males
Chil3	x						Yes	Yes	Yes
Pla2g4a	x	x					Yes	Yes	Yes
Slc9b2				x			Yes	Yes	Yes
Bdh2		x					Yes	Yes	No
Slc26a7				x			Yes	Yes	No
Slc6a14				x			Yes	Yes	No
Cd28	x						Yes	No	No
Rasgrp1	x						Yes	No	Yes
Tgtp1	x						Yes	No	No
Hamp	x			x			Yes	No	Yes
Il1a	x						Yes	No	Yes
Snhg1	x						Yes	No	Yes
Apold1		x	x		x	x	Yes	No	Yes
Cyb5r2		x					Yes	No	Yes
Gdpd3		x					Yes	No	No

Hpgds		x					Yes	No	Yes
Acsbg1		x					Yes	No	Yes
Acsm3		x					Yes	No	No
Nox4			x				Yes	No	No
Gucy1a2			x				Yes	No	No
Ptprn			x				Yes	No	No
Rgs4				x			Yes	No	Yes
Slc10a6				x			Yes	No	No
Fgf9						x	Yes	No	No
Slc30a4				x			No	Yes	No
Slc9b1				x			No	Yes	No
Oas1b	x						No	No	Yes
Clec2d	x						No	No	Yes
Gdap10	x						No	No	Yes
Il18bp	x						No	No	Yes
Lgals4	x						No	No	Yes
Pisd-ps1	x						No	No	Yes
Snhg20	x						No	No	Yes
Alms1		x					No	No	Yes
Echdc2		x					No	No	Yes
Phka2		x					No	No	Yes
Gabre				x			No	No	Yes
Gabrr2				x			No	No	Yes
Atg9b					x		No	No	Yes
Spx					x		No	No	Yes

Table S4. Genes differentially expressed by a HFHS diet for overall, female-specific and male-specific comparisons that are located on the X chromosome.

Gene ID	Gene Symbol	Chromosome	Direction of Change	Comparison
ENSMUSG00000031380	Vegfd	X:164373378-164402650	downregulated	Overall
ENSMUSG00000051228	Nyx	X:13466110-13489313	downregulated	Overall
ENSMUSG00000059327	Eda	X:99975606-100400762	downregulated	Overall
ENSMUSG00000087149	Itih5l-ps	X:150826972-150857053	downregulated	Overall
ENSMUSG00000031089	Slc6a14	X:21714896-21742355	upregulated	Overall
ENSMUSG00000031163	Glod5	X:8004200-8018492	upregulated	Overall
ENSMUSG00000073062	Zxdb	X:94724569-94730187	upregulated	Overall
ENSMUSG00000051228	Nyx	X:13466110-13489313	downregulated	Females
ENSMUSG00000059327	Eda	X:99975606-100400762	downregulated	Females
ENSMUSG00000031089	Slc6a14	X:21714896-21742355	upregulated	Females
ENSMUSG00000079584	Gm364	X:57409154-57488767	upregulated	Females
ENSMUSG00000031200	Mtcp1	X:75410444-75416588	upregulated	Males
ENSMUSG00000031295	Phka2	X:160502166-160598878	upregulated	Males
ENSMUSG00000031303	Map3k15	X:159988433-160123351	upregulated	Males
ENSMUSG00000031340	Gabre	X:72255999-72274803	upregulated	Males
ENSMUSG00000031362	Xlr4c	X:73233688-73243130	upregulated	Males
ENSMUSG00000031380	Vegfd	X:164373378-164402650	downregulated	Males
ENSMUSG00000034160	Ogt	X:101640060-101684351	upregulated	Males
ENSMUSG00000055653	Gpc3	X:52272426-52613950	downregulated	Males
ENSMUSG00000059327	Eda	X:99975606-100400762	downregulated	Males
ENSMUSG00000067768	Xlr4b	X:73214333-73222453	upregulated	Males
ENSMUSG00000073125	Xlr3b	X:73192207-73202930	upregulated	Males
ENSMUSG00000085396	Firre	X:50555744-50635321	upregulated	Males
ENSMUSG00000101603	Gm28730	X:53051194-53053159	upregulated	Males

Table S5. Genes differentially expressed by a HFHS diet compared to an intrauterine inflammation model.

Overlapped DEGs from Lien et al. 2020, and their direction of change. Genes differentially expressed in the placenta by a maternal HFHS diet (overall, female and male-specific comparisons) overlapped with the list of genes differentially expressed in response to intrauterine inflammation (50).

Comparison	Gene	Lien et al. 2020		Candia et al. 2023	
		**LogFC**	Direction of change	LogFC	Direcction of change
Overal	Apoa2	0.4676	Upregulated	-1.664	Downregulated
	Derl3	-0.2909	Downregulated	-0.8214	Downregulated
	Hnf4a	0.2383	Upregulated	-0.6725	Downregulated
	Igfbp1	0.1945	Upregulated	-0.8751	Downregulated
	Endou	-0.2226	Downregulated	-0.6593	Downregulated
	Igfbp6	-0.4652	Downregulated	-0.9445	Downregulated
	Scd2	0.3195	Upregulated	-0.8336	Downregulated
	Ccdc184	1.249	Upregulated	-0.5979	Downregulated
	Elovl4	-0.2856	Downregulated	-0.8061	Downregulated
	Abtb2	0.6324	Upregulated	-0.5853	Downregulated
	Spata2l	-0.7153	Downregulated	-0.6037	Downregulated
	Cdhr2	0.4082	Upregulated	-1.981	Downregulated
	Thrsp	-0.5856	Downregulated	-0.749	Downregulated
	Cysrt1	-0.308	Downregulated	-0.677	Downregulated
	Asb10	0.3591	Upregulated	-0.6987	Downregulated
	Pcp4l1	-0.3033	Downregulated	-0.7501	Downregulated
	Unc5cl	0.395	Upregulated	-0.9061	Downregulated

	Rarres1	-0.2644	Downregulated	-0.6824	Downregulated
	Spink2	0.0994	Upregulated	-0.7265	Downregulated
	Chst8	0.3231	Upregulated	-0.5981	Downregulated
	Itih5l-ps	-0.3358	Downregulated	-0.7817	Downregulated
	Gm6967	-0.4597	Downregulated	-0.8069	Downregulated
	Adora2b	1.2272	Upregulated	0.5969	Upregulated
	4930412O13Rik	0.3467	Upregulated	0.8344	Upregulated
	Il1a	1.6012	Upregulated	0.9643	Upregulated
	Slc10a6	0.6251	Upregulated	0.7399	Upregulated
	Ing4	-0.2686	Downregulated	0.7233	Upregulated
	Gdpd3	1.0079	Upregulated	2.972	Upregulated
	Slc6a14	0.3296	Upregulated	0.5905	Upregulated
	Rgs4	0.6455	Upregulated	0.6297	Upregulated
	Chil3	-0.5241	Downregulated	1.416	Upregulated
	Samd9l	1.2516	Upregulated	0.6415	Upregulated
	Hamp	1.1941	Upregulated	1.18	Upregulated
	Tgtp1	1.321	Upregulated	0.9841	Upregulated
	Gm15972	-0.4264	Downregulated	0.6378	Upregulated
	Apold1	0.9598	Upregulated	0.8594	Upregulated
	Gm20627	-0.373	Downregulated	0.7379	Upregulated
	D5Ertd605e	-0.3257	Downregulated	0.6978	Upregulated
Females	Scd2	0.3195	Upregulated	-0.635	Downregulated
	Thrsp	-0.5856	Downregulated	-0.9754	Downregulated
	Pnoc	-0.2869	Downregulated	-2.585	Downregulated
	Slc6a14	0.3296	Upregulated	0.7507	Upregulated
	Chil3	-0.5241	Downregulated	1.268	Upregulated
Males	Ltbp1	0.3008	Upregulated	-0.6296	Downregulated
	Htra1	0.3239	Upregulated	-0.6936	Downregulated
	Slc5a1	0.3765	Upregulated	-1.662	Downregulated

	Slc9a5	0.3937	Upregulated	0.6846	Upregulated
	Wnt7b	-0.2844	Downregulated	-0.6233	Downregulated
	Endou	-0.2226	Downregulated	-0.6909	Downregulated
	Igfbp6	-0.4652	Downregulated	-0.9746	Downregulated
	Pgc	-0.092	Downregulated	-0.6118	Downregulated
	Zfp397	-0.3159	Downregulated	0.6907	Upregulated
	Cpn1	0.3036	Upregulated	-1.923	Downregulated
	Scd2	0.3195	Upregulated	-0.9842	Downregulated
	4930412O13Rik	0.3467	Upregulated	1.449	Upregulated
	Il1a	1.6012	Upregulated	1.345	Upregulated
	Echdc2	0.4017	Upregulated	0.6956	Upregulated
	Oas1b	0.7805	Upregulated	0.7144	Upregulated
	1600015I10Rik	-0.5099	Downregulated	0.8993	Upregulated
	Trpv6	-0.2448	Downregulated	-0.7122	Downregulated
	Clec2d	0.3596	Upregulated	0.6639	Upregulated
	Ing4	-0.2686	Downregulated	1.082	Upregulated
	Syt8	0.2537	Upregulated	0.7586	Upregulated
	Elovl4	-0.2856	Downregulated	-0.895	Downregulated
	Uba7	0.9647	Upregulated	0.951	Upregulated
	Ppip5k1	0.2791	Upregulated	0.7134	Upregulated
	Fam208b	0.2572	Upregulated	0.5851	Upregulated
	Ptgdr2	-0.5465	Downregulated	-0.603	Downregulated
	Cdhr2	0.4082	Upregulated	-2.572	Downregulated
	Creb3l3	0.3708	Upregulated	-1.15	Downregulated
	Cysrt1	-0.308	Downregulated	-0.6707	Downregulated
	Asb10	0.3591	Upregulated	-0.8802	Downregulated
	Rgs4	0.6455	Upregulated	0.7965	Upregulated
	A230050P20Rik	0.6014	Upregulated	0.6085	Upregulated
	Bace2	0.2868	Upregulated	-0.7172	Downregulated

	Chil3	-0.5241	Downregulated	1.599	Upregulated
	2010003K11Rik	0.1909	Upregulated	-2.457	Downregulated
	Lgals2	0.2674	Upregulated	-1.132	Downregulated
	Pnoc	-0.2869	Downregulated	-1.972	Downregulated
	Samd9l	1.2516	Upregulated	0.8596	Upregulated
	Rarres1	-0.2644	Downregulated	-0.7834	Downregulated
	Hamp	1.1941	Upregulated	1.234	Upregulated
	Cx3cr1	-0.2885	Downregulated	-0.7852	Downregulated
	Spink2	0.0994	Upregulated	-0.8786	Downregulated
	2010109I03Rik	0.5219	Upregulated	0.7922	Upregulated
	Hsp25-ps1	0.8689	Upregulated	-0.6478	Downregulated
	Pisd-ps1	-0.5627	Downregulated	0.6441	Upregulated
	Apold1	0.9598	Upregulated	0.947	Upregulated
	Gm20627	-0.373	Downregulated	0.9368	Upregulated
	Gdap10	0.318	Upregulated	1.103	Upregulated
	Gm4673	-0.3378	Downregulated	0.6196	Upregulated

Table S6. Genes differentially expressed by a HFHS diet related to biological processes of interest compared to an intrauterine inflammation model.

Overlapped DEGs from Lian et al. 2020 and Candia et al. 2023, and their direction of change related to biological process of interest.

Comparison	Gene	Lien 2020		Candia 2023	
		**LogFC**	Direction of change	LogFC	Direcction of change
Overall	**Il1a**	1.6012	Upregulated	0.9643	Upregulated
	**Slc10a6**	0.6251	Upregulated	0.7399	Upregulated
	**Gdpd3**	1.0079	Upregulated	2.972	Upregulated
	**Slc6a14**	0.3296	Upregulated	0.5905	Upregulated
	**Rgs4**	0.6455	Upregulated	0.6297	Upregulated
	Chil3	-0.5241	Downregulated	1.416	Upregulated
	**Hamp**	1.1941	Upregulated	1.18	Upregulated
	**Tgtp1**	1.321	Upregulated	0.9841	Upregulated
	**Apold1**	0.9598	Upregulated	0.8594	Upregulated
	**Elovl4**	-0.2856	Downregulated	-0.8061	Downregulated
	**Thrsp**	-0.5856	Downregulated	-0.749	Downregulated
	Unc5cl	0.395	Upregulated	-0.9061	Downregulated
	Scd2	0.3195	Upregulated	-0.8336	Downregulated
Females	**Slc6a14**	0.3296	Upregulated	0.7507	Upregulated
	Chil3	-0.5241	Downregulated	1.268	Upregulated
	Scd2	0.3195	Upregulated	-0.635	Downregulated
	**Thrsp**	-0.5856	Downregulated	-0.9754	Downregulated
Males	**Il1a**	1.6012	Upregulated	1.345	Upregulated
	**Echdc2**	0.4017	Upregulated	0.6956	Upregulated
	**Oas1b**	0.7805	Upregulated	0.7144	Upregulated
	**Clec2d**	0.3596	Upregulated	0.6639	Upregulated

	**Rgs4**	0.6455	Upregulated	0.7965	Upregulated
	Chil3	-0.5241	Downregulated	1.599	Upregulated
	**Hamp**	1.1941	Upregulated	1.234	Upregulated
	Pisd-ps1	-0.5627	Downregulated	0.6441	Upregulated
	**Apold1**	0.9598	Upregulated	0.947	Upregulated
	**Gdap10**	0.318	Upregulated	1.103	Upregulated
	**Wnt7b**	-0.2844	Downregulated	-0.6233	Downregulated
	Scd2	0.3195	Upregulated	-0.9842	Downregulated
	**Trpv6**	-0.2448	Downregulated	-0.7122	Downregulated
	**Elovl4**	-0.2856	Downregulated	-0.895	Downregulated
	Lgals2	0.2674	Upregulated	-1.132	Downregulated
	**Cx3cr1**	-0.2885	Downregulated	-0.7852	Downregulated
